# The eukaryotic MEP-pathway genes are evolutionarily conserved and originated from Chlaymidia and cyanobacteria

**DOI:** 10.1186/s12864-021-07448-x

**Published:** 2021-02-26

**Authors:** Liping Zeng, Katayoon Dehesh

**Affiliations:** Institute for Integrative Genome Biology and Department of Botany and Plant Sciences, University of California, Riverside, CA 92521 USA

**Keywords:** Isoprenoid, MEP-pathway, Plastid-bearing eukaryotes, Phylogenetic, Polyphyletic

## Abstract

**Background:**

Isoprenoids are the most ancient and essential class of metabolites produced in all organisms, either via mevalonate (MVA)-and/or methylerythritol phosphate (MEP)-pathways. The MEP-pathway is present in all plastid-bearing organisms and most eubacteria. However, no comprehensive study reveals the origination and evolutionary characteristics of MEP-pathway genes in eukaryotes.

**Results:**

Here, detailed bioinformatics analyses of the MEP-pathway provide an in-depth understanding the evolutionary history of this indispensable biochemical route, and offer a basis for the co-existence of the cytosolic MVA- and plastidial MEP-pathway in plants given the established exchange of the end products between the two isoprenoid-biosynthesis pathways. Here, phylogenetic analyses establish the contributions of both cyanobacteria and Chlamydiae sequences to the plant’s MEP-pathway genes. Moreover, Phylogenetic and inter-species syntenic block analyses demonstrate that six of the seven MEP-pathway genes have predominantly remained as single-copy in land plants in spite of multiple whole-genome duplication events (WGDs). Substitution rate and domain studies display the evolutionary conservation of these genes, reinforced by their high expression levels. Distinct phenotypic variation among plants with reduced expression levels of individual MEP-pathway genes confirm the indispensable function of each nuclear-encoded plastid-targeted MEP-pathway enzyme in plant growth and development.

**Conclusion:**

Collectively, these findings reveal the polyphyletic origin and restrict conservation of MEP-pathway genes, and reinforce the potential function of the individual enzymes beyond production of the isoprenoids intermediates.

**Supplementary Information:**

The online version contains supplementary material available at 10.1186/s12864-021-07448-x.

## Background

**Fig. 1:**
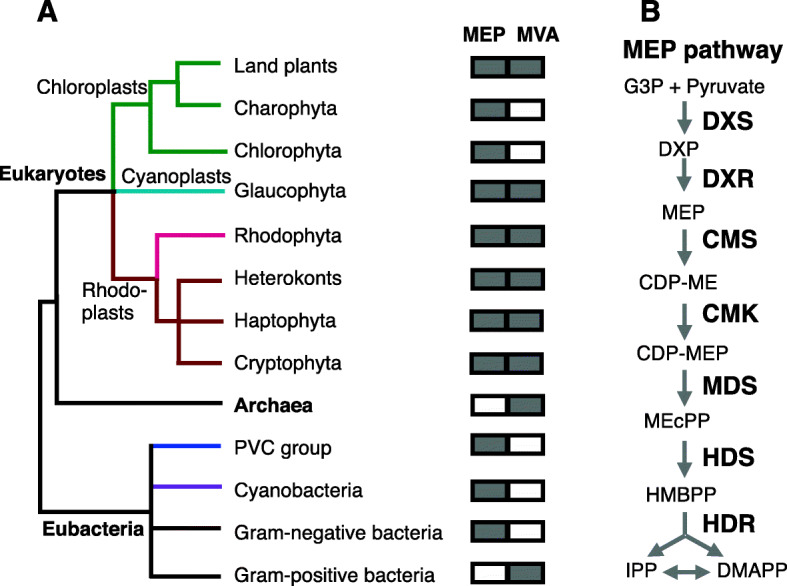
Distribution of isoprenoid biosynthesis-pathways across lineages. **a** Distribution of mevalonate (MVA) and/or methylerythritol dicyclophosphate (MEP) pathways, the two isoprenoid biosynthesis-pathways across linages of eukaryotes, Achaea, and eubacteria. The rectangular boxes display the presence (filled) or absence of (blank) MVA- or MEP-pathway in each of the lineages. **b** Schematic presentation of the seven enzymes of the MEP-pathway producing the two universal five-carbon precursors, isopentenyl diphosphate (IPP) and its isomer dimethylallyl diphosphate (DMAPP). DXS: 1-deoxy-D-xylulose-5-phosphate synthase. DXR: 1-deoxy-D-xylulose 5-phosphate reductoisomerase. CMS: 4-Diphosphocytidyl-2C-methyl-D-erythritol synthase. CMK: 4-(cytidine 5′-diphospho)-2-C-methyl-D-erythritol kinase activity. MDS: 2C-methyl-d-erythritol 2, 4-cyclodiphosphate synthase. HDS: 4-hydroxy-3-methylbut-2-enyl diphosphate synthase. HDR: and 4-hydroxy-3-methylbut-2-en-1-yl diphosphate reductase

With over 55,000 molecules, isoprenoids are the most ancient group of structurally and functionally diverse metabolites essential for all kingdoms of life [1]. Isoprenoid-derived compounds in free-living organisms range from hormones, lipids, pigments, vitamins, electron transport chain and defense compounds, and as such of industrial interests for drugs, agrochemicals, rubber and fragrances [2]. However, despite their diversity, isoprenoids are derived from two universal five-carbon precursors, isopentenyl diphosphate (IPP) and its isomer dimethylallyl diphosphate (DMAPP) [3]. These precursors are synthesized either by mevalonate (MVA)-pathway [4] and/or by the alternative route methyl erythritol phosphate (MEP)-pathway [5]. Almost all eukaryotes, archae and some gram-positive bacteria employ the MVA-pathway, whereas most gram-negative bacteria, cyanobacteria and green algae exclusively use MEP-pathway (Fig. 1a) [6]. Plastid-bearing eukaryotes however are unique as they have retained both pathways compartmentalized in the cytosol (MVA) and plastids (MEP) [2]. It is suggested that retention of the two pathways in the two different subcellular compartments of the plastid-bearing eukaryotic cell is to regulate isoprenoid biosynthesis according to the availability of carbon and energy currencies, and as a strategy to balance resource allocation between growth and adaptive responses to unfavorable environmental inputs [7]. Given the established metabolic exchanges between MVA- and MEP-pathway in higher plants [8–10], the biological grounds for the indispensable function of each of these pathways in plants has remained an enigma.

One of the most profound outcome of evolution is the emergence of plastids through a single endosymbiotic event accompanied by a complex mix of loss, movement and replacement in the ancestor of eukaryotes [11]. The endosymbiotic events that led to the origination of plastids were ensued by the transfer of genetic material from the endosymbiont to the nuclear genome of the host, followed by the evolution of protein import machinery for transferring nuclear-encoded plastid-targeted proteins and by extension the inevitable establishment of plastids-to-nucleus (retrograde signaling) signaling cascades [12, 13]. The retrograde signaling cascade is instrumental for coordination of vital activities between the two subcellular genomes in plastid-bearing eukaryotes.

One essential plastidial biochemical route is the MEP-pathway, responsible for catalyzing glyceraldehyde 3-phosphate and pyruvate into isopentenyl diphosphate (IPP) and dimethylallyl diphospahte (DMAPP), the central intermediates in the biosynthesis of isoprenoids (Fig. 1b). The MEP-pathway is comprised of seven nuclear genes encoding plastid-localized enzymes. Intriguingly, one of the MEP-pathway intermediates, MEcPP (2-C-methyl-D-erythritol-2, 4-cyclopyrophosphate), is found to be a bi-functional entity severing as a precursor of isoprenoids and as a stress-specific retrograde signaling metabolite coordinating expression of selected stress-response nuclear genes [14, 15].

Given the antiquity and essential function of isoprenoids, the evolutionary history of the MVA-pathway in eukaryotes is extensively examined [2, 6] whereas, the characteristics of MEP-pathway genes is thus far restricted to limited species and as such incomplete [16]. Understanding the evolutionary history of the MEP-pathway across a wide range of species offers a novel insight into their contribution to the evolution of primary plastids.

Here, extensive and integrated phylogenetic analyses identify alpha-proteobacteria, cyanobacteria and Chlamydiae as the bacterial lineages that have contributed to the evolution of MEP-pathway genes in plastid-bearing eukaryotes. Syntenic analyses establish the predominant presence of MEP-pathway genes as single-copy resulted from the loss of duplicated copies post whole genome duplication (WGD) events in land plants. Inter-species syntenic block and substitution rate analyses reveal the evolutionarily conservation of the MEP-pathway genes. Moreover, genetics analyses establish essential but differential functions of the MEP-pathway enzymes in plant growth and development.

In summary, the finding uncovers the evolutionary history and characteristics of plastidial isoprenoid biosynthesis-pathway genes, and reinforces the uniqueness of the MEP-pathway for unmasking the origins and evolution of plastids.

## Results

### MEP-pathway genes in plastid-bearing eukaryotes are derived from different bacteria lineages

**Fig. 2:**
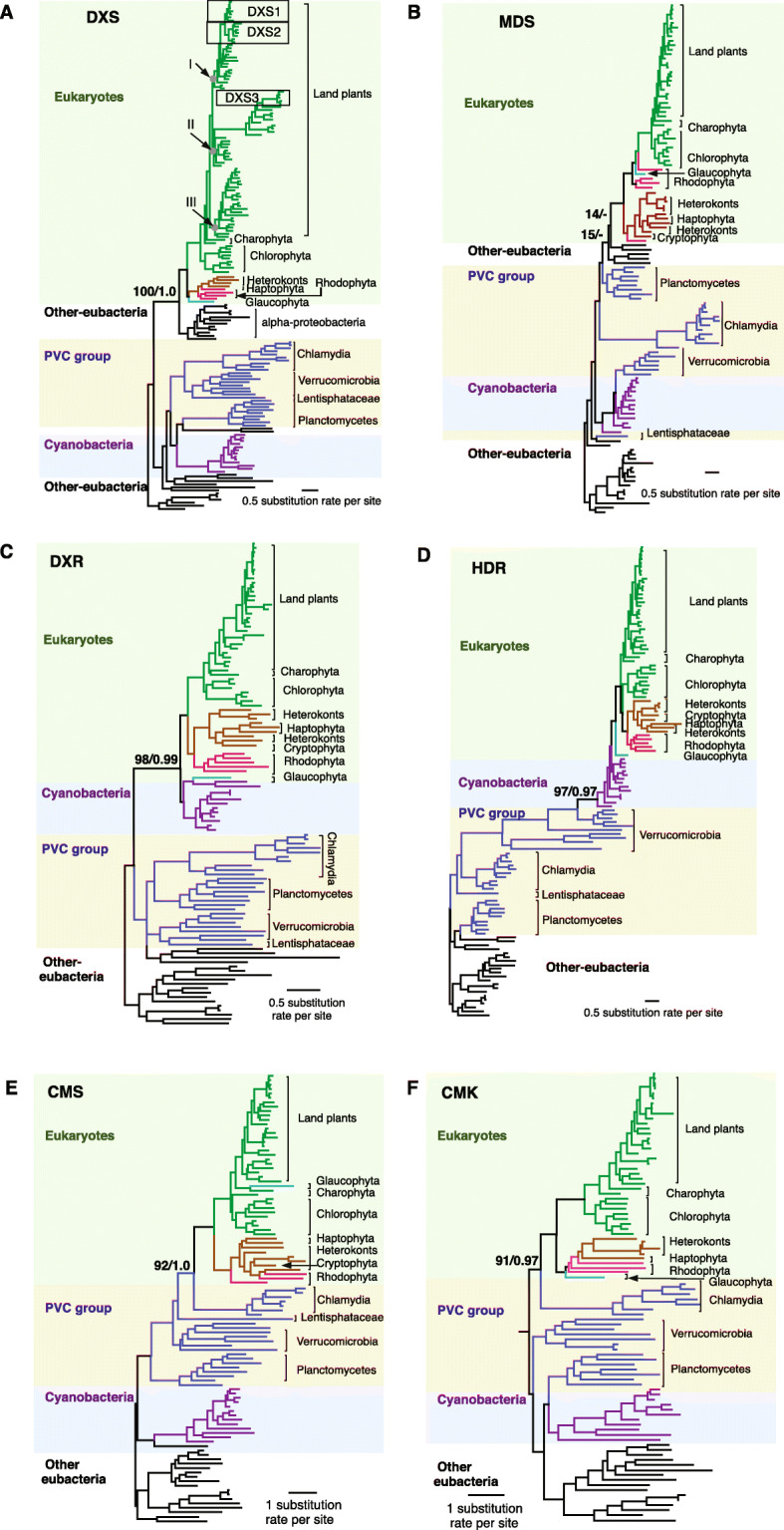
Origin of plastid-bearing eukaryotes MEP-pathway genes. The cladograms display clustering of plastid-bearing eukaryotes DXS (**a**), MDS (**b**), DXR (**c**), HDR (**d**), CMS (**e**) and CMK (**f**) with other-eubacteria, obtained from RAxML using amino acid sequences. Taxa in various major groups are shown in different colors. Species in eukaryotes, PVC groups and Cyanobacteria are shaded with light green, light yellow and light blue, respectively. Numbers associated with branches are bootstrap (BS) values obtained from RAxML and posterior probability obtained from MrBayes. The dash associated with the branches of eukaryotes and the other-eubacteria shown as 14/− and 15/−indicate that the relationships are not supported by MrBayes

**Fig. 3:**
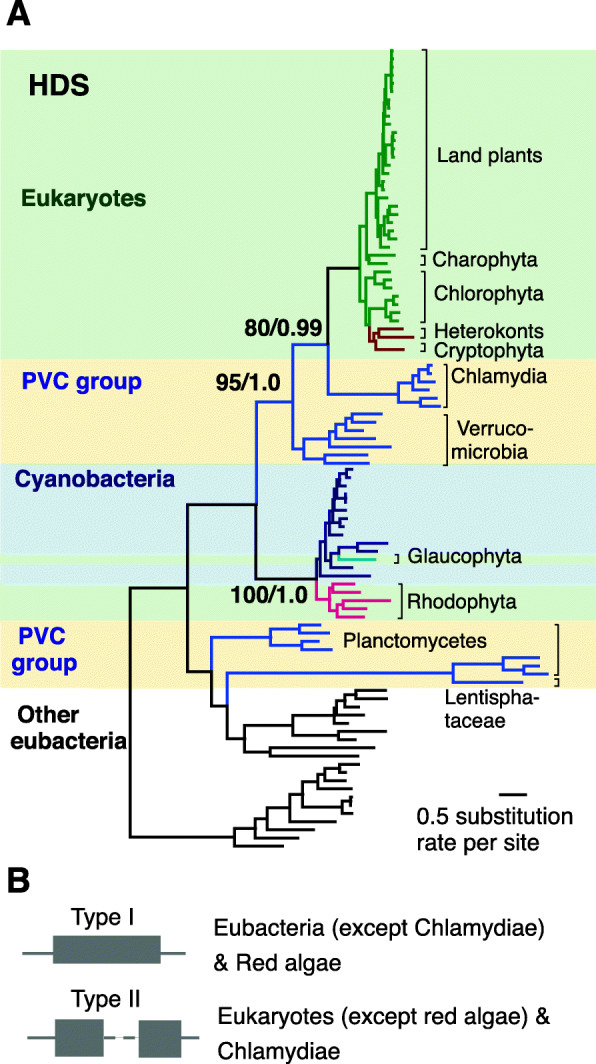
Origin and domain structure of HDS. **a** Cladograms cluster plastid-bearing eukaryotes HDS with chlamydia amino acid sequences. The cladograms are obtained from RAxML using amino acid sequences. Taxa in different major groups are shown in different colors. Species in plastid-bearing eukaryotes, PVC groups and Cyanobacteria are shaded with light green, light yellow and light blue, respectively. Numbers associated with branches are bootstrap (BS) values obtained from RAxML and posterior probability are obtained from MrBayes. **b** Schematics of the types (type I and type II) of gcpE domain in HDS enzyme and their presence in their corresponding species

To gain insight into the evolution of the MEP-pathway genes, we constructed phylogenetic trees for individual genes by using protein sequences of a wide range of species from eukaryotes, cyanobacteria, PVC (Planctomycetes, Verrucomicrobia and Chlamydiae) group bacteria, and other non-cyanobacteria and non-PVC group bacteria (hereafter named them as ‘other-eubacteria’). These analyses reveal the multiple origins of MEP-pathway genes in plastid-bearing eukaryotes (Figs. 2 a-f, 3 and S1-S7).

The phylogenetic tree analyses show *DXS* and *MDS* in plastid-bearing eukaryotes and other-eubacteria are sister groups. It is of note that while *DXS* in plastid-bearing eukaryotes is clearly derived from alpha-proteobacteria (Figs. 2a and S1), the specific inheritance source for *MDS* remains unclear (Figs. 2b and S2). The maximum likelihood tree of *MDS* moderately supports *Aquifex Aeolicus* and *Leptospira interrogans* as the closest relatives of plastid-bearing eukaryotes (Fig. S2A), whereas, the Bayesian tree clusters Deinococcus-Thermus bacteria (*Thermus thermophiles, Deinococcus radiodurans*) with plastid-bearing eukaryotes (Fig. S2B).

The phylogenetic trees of *DXR* and *HDR* group eukaryotes sequences with cyanobacteria (Figs. 2 c-d, and S3-S4), and eukaryotic CMS and CMK in cluster with Chlamydiae (Figs. 2 e-f and S5-S6).

Interestingly, the phylogenetic tree of *HDS* separates the plastid-bearing eukaryotes into two clades; one clade clusters with Chlamydiae and the other is closest to the cyanobacteria homologue (Figs. 3a and S7). Moreover, protein structure analyses show that depending on the organism, HDS enzymes have two different types of gcpE domains. Eubacteria HDS contain type I gcpE domain comprised of two N- and C-terminal parts, whereas the type II domain present in plants contains an additional domain between the N- and C-terminal parts of the protein, thought to enable the enzyme to function as a monomer (Fig. 3b) [17]. Domain analyses identify red algae HDS as an eubacteria-like type I-enzyme rather than the expected type II-enzyme in eukaryotes, while Chlamydiae HDS possesses the type II domain instead of the expected eubacterial type I domain (Fig. 3b).

Collectively the results display contributions of different bacterial lineages to the origins of MEP-pathway genes in plastid-bearing eukaryotes.

### Duplicated *DXS* copies are not functionally redundant

Among the seven MEP-pathway enzymes, DXS catalyzes the first step in isoprenoid biosynthesis [18] (Fig. 1b). Phylogenetic analyses of DXS show the presence of one gene copy in examined algae and eubacteria, and its expansion into three subfamilies (I to III) in land plants (Figs. 2a and S1).

In the subfamily I, gene duplications in each common ancestor of Brassicaceae (Cruciferae, e.g. cabbage and turnip) and Fabaceae (legume, e.g. soybean) resulted in the presence of two genes (*DXS1* and *DXS2*). In the subfamily II, there is only one *DXS* copy, designated *DXS3* in *Arabidopsis thaliana*, Brassicaceae and the ancestor of Fabaceae. Moreover, the subfamily II is absent in gymnosperms, but duplicated copies are present in several moss and lycophyte species. Strikingly, the subfamily III branch is lost in Brassicaceae family, whereas Fabaceae and grape display species-specific duplication(s), and gymnosperms maintain two copies of subfamily III in their ancestor.

Interestingly, despite of the presence of three DXS subfamilies in land plants, only one is reported to function as a housekeeping MEP-pathway gene, such as *DXS1* in *A. thaliana* that encodes the functional MEP-pathway enzyme [19]. This is supported by plastidial localization of DXS1 in Fabaceae species *Medicago* and soybean, in line with the function of the enzyme catalyzing the first step of the MEP-pathway [20, 21]. The DXS2, which has no DXS activity, is assumed to synthesize specific isoprenoids related to mycorrhiza in *Medicago* [22]. DXS3, as the most divergent member of the family, has the expected DXS enzyme activity, but is expressed at very low levels, provoking the idea of its involvement in the synthesis of phytohormones in maize [23]. It is of note that in *Escherichia coli* DXS is responsible for production of vitamin B6, but this synthesis in plants utilizes intermediates of the glycolytic and pentose phosphate pathways rather than that of DXS [24]. This information eliminates the possible function of plant’s additional DXSs in vitamin B6 production.

In summary, despite the preservation of duplicated *DXS* copies in land plants, only one copy has retained the ancestral function of catalyzing the first step of the MEP-pathway, alluding to a possible loss or neo-functionalization of additional copies.

### MEP-pathway genes are predominantly single copies

WGD events are most prevalent during angiosperm’s diversity and are found in the common ancestors of seed plants [25]. However in spite of gene duplication events there are ~ 3124 nuclear-encoded single-copy genes, comprising ~ 11% of Arabidopsis genome, shared by other angiosperms [26]. Excluding *DXS*, and with exception of few species that experienced a recent WGD, such as soybean and *Brassica oleracea* (Figs. S2-S7), the remainder six MEP-Pathway genes are among the single-copy genes in all algae and most land plants. Even in exceptional cases of soybean and *Brassica oleracea*, the MEP-pathway gene such as *CMS* remained as single copy (Fig. S5).

**Fig. 4:**
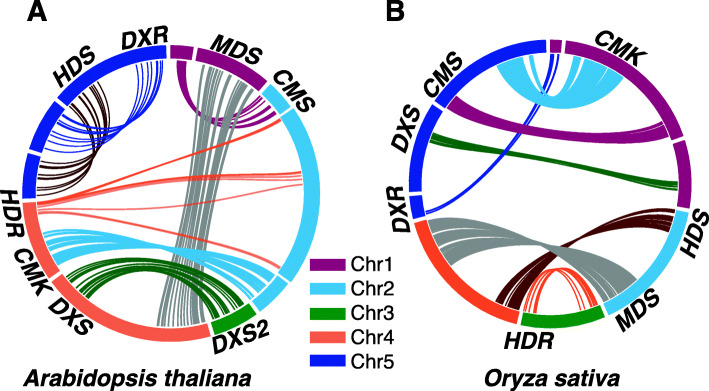
Paralogous syntenic blocks display loss of MEP-pathway duplicated copies. Arabidopsis (**a**) and rice (**b**) genes presented in the same colored bands are pairs of paralogues. The band weight correlates to gene numbers on chromosome fragments depicted in different colors

Despite multiple WGD events, the predominant presence of MEP-pathway genes as single-copy in most land plants leads to the question of when the duplicated copies were lost. To address this, we constructed intra-species conserved syntenic blocks of MEP-pathway genes for *A. thaliana* and *Oryza sativa*, the model eudicot and monocot species respectively. Both species are current diploids even though their most recent common ancestors experienced two WGDs. Except for the *DXS* in *A. thaliana*, the MEP-pathway genes in both species are single-copy, separately positioned in a syntenic block surrounded by pairs of paralogs retained after WGD(s) (Fig. 4 and Data S1). This data supports the notion of loss of MEP-pathway genes post WGD.

To gain insight into the fate of the ‘lost’ copy of MEP-pathway genes, we searched for remnants of duplication events, but found no evidence such as the presence of a pseudogene for any of the MEP-pathway genes in *A. thaliana* genome.

### MEP-pathway genes are evolutionarily conserved

To investigate the evolutionary characteristics of MEP-pathway genes, we examined the evolutionary rate, and domain architectures of the encoded proteins.

**Fig. 5:**
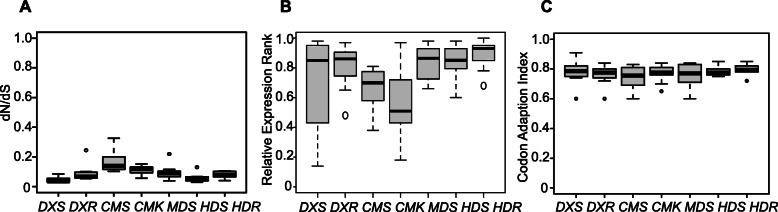
MEP-pathway genes evolved slowly and are highly expressed. **a** The dN/dS ratios of MEP-pathway genes. **b** The relative expression ranking of MEP-pathway genes. **c** The Codon Adaption Index (CAI) of MEP-pathway genes. All data are displayed as scatter-boxplots with the maximum value of 1.0

The evolutionary divergence of DNA can be estimated by the ratio of substitution rates at non-synonymous (dN; amino acid altering) and synonymous (dS; silent) sites, a measure of the dynamics of molecular evolution [27]. That is, a significantly low ratio of dN/dS marks slow evolution and as such the conserved nature of the protein. To investigate the MEP-pathway genes’ evolutionary rates we examined their respective dN/dS ratios in selected species from represented lineages of plastid-bearing eukaryotes. The markedly low dN/dS median values ranging 0.04–0.14 suggests a strong purification selection for all the seven MEP-pathway genes, thereby supporting their evolutionary conservation (Fig. 5a and Table S1).

Moreover the analyses of the protein domain(s) structure of MEP-pathway enzymes establishes that, with the exception of HDS, an enzyme with two different types of gcpE domains (Fig. 3b), the protein structures of the remainder of MEP-pathway enzymes are universally conserved [28].

### MEP-pathway genes are highly expressed

There are two theories regarding gene conservation as the result of evolutionary rate of proteins; i) an inverse relationship between the expression levels and the evolutionary rate [29]; and ii) a slow evolution of functionally critical genes as opposed to less critical ones [30]. To test the potential contribution of these two scenarios to the high conservation of the MEP-pathway genes, we obtained and ranked expression levels of all MEP-pathway genes by analyzing the publicly available genome-wide transcriptomic datasets of representative land species, such as eudicots (*A. thaliana* and soybean), monocots (*O. sativa* and *Zea mays*), gymnosperm (*Picea abies*), moss (*P. patens*) and lycophyte (*S. moellendorffii*). The data illustrate high expression levels for most MEP-pathway genes with the exception of the three duplicated copies of *DXS* and two duplicates of *HDS* in *P. patens*, and one duplicated copy of *CMK* in soybean (Fig. 5b and Table S2). Notably, in most species, expression-ranking data places the first two genes (*DXS* and *DXR*) and the last three genes (*MDS*, *HDS* and *HDR*) amongst the top 5–10% most abundant transcripts.

To compensate for the absence of transcriptomic datasets for several lineages, we recruited a widely used quantitative method, Codon Adaptation Index (CAI), to predict the expression level of a gene based on its codon sequence. The rationale of CAI is based on codon degeneracy, and that the highly expressed genes are biased towards the codon decoded by the most abundant tRNA species [31]. We therefore calculated CAIs of all MEP-pathway genes from represented species with and without transcriptomic datasets in all life lineages. In most analyzed species, the MEP-pathway genes have a CAI value higher than 0.7 (Table S3). The median CAI values for the MEP-pathway genes (0.76–0.80), denote their high expression levels in all life lineages analyzed (Fig. 5c).

In summary, the high expression levels of the MEP-pathway genes support their evolutionary conservation.

### MEP-pathway genes are indispensable for plant growth

Except green algae, plants possess both the cytosolic MVA- and the plastidial MEP-pathways, despite the established exchanges of the end products between the two isoprenoid producing routes [10]. Given the indispensable function of MEP-pathway genes in eubacteria [32, 33], we employed genetic approaches to test the likelihood of the essentiality of the MEP-pathway genes in plants.

**Fig. 6:**
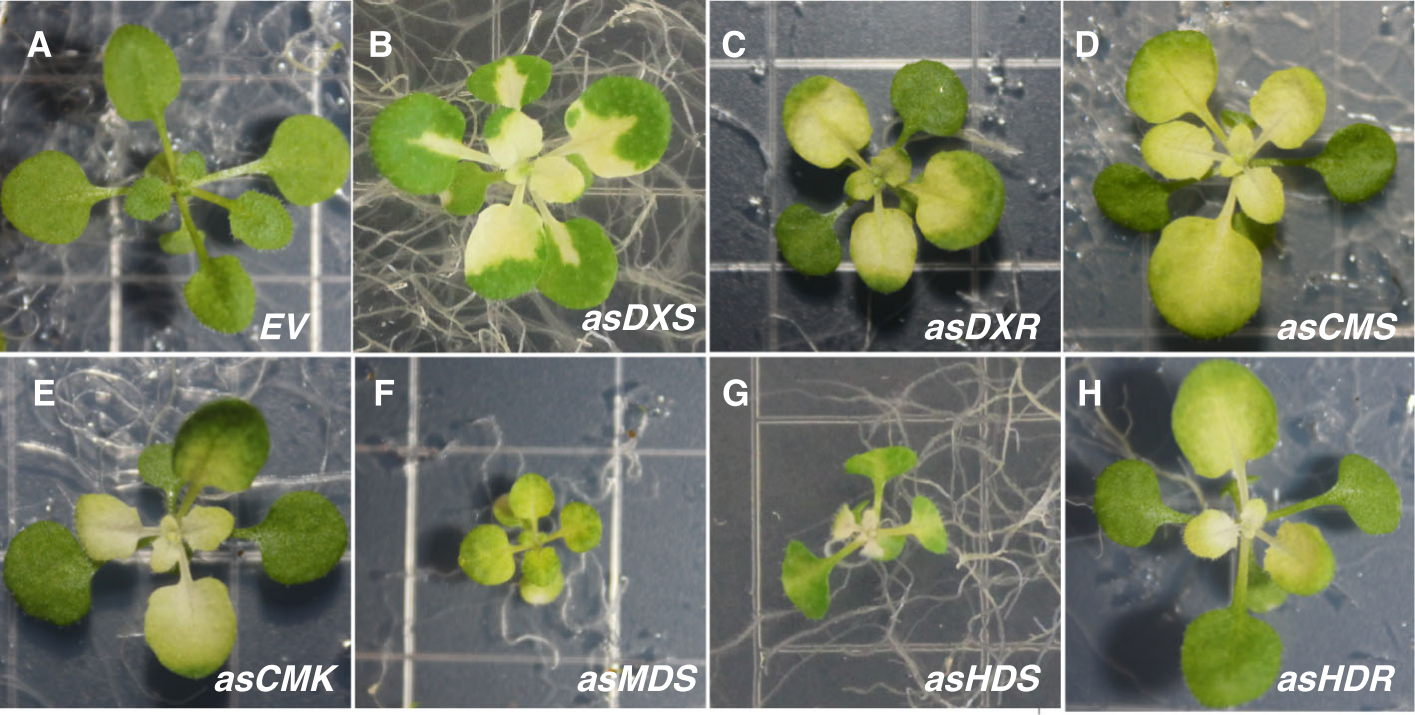
The MEP-pathway genes are indispensable for plant growth. Representative images of 2-weeks-old seedlings of RNAi lines and wild type transformed with empty vector (EV)

Unavailability of T-DNA insertion lines for the MEP-pathway genes, led us to employ the previously generated RNAi lines that were maintained as segregating population for individual MEP-pathway genes in *A. thaliana* [15]. Homozygous RNAi lines, each with 92–95% reduced expression levels of the corresponding MEP-pathway genes [15], displayed seedling size and variegation leaf phenotypes distinct from each other and from those of the wild type plants transformed with an empty vector (EV). These visibly altered phenotypes include dwarfed stature of *asDXR*, *asMDS* and *asHDS* lines; in concert with pale-yellow leaves phenotype of the *asMDS* seedlings, and an albino phenotype of true leaves in all the other six RNAi lines (Fig. 6).

In summary, the phenotypes of RNAi lines confirm the indispensable function of MEP-pathway enzymes in plant growth and development, and that the markedly different size and phenotypic characteristics of each line suggest the involvement of these enzymes in distinct functions in addition of their role as intermediates in isoprenoids biosynthesis pathway.

## Discussion

The MEP-pathway is comprised of seven nuclear-encoded plastid-localized enzymes, essential for plant growth and key to stress-specific retrograde signaling as evidenced by the function of the MEP-pathway intermediate, methylerythritol cyclodiphosphate (MEcPP) as a retrograde signaling metabolite [15]. The retrograde signaling function of MEcPP offers an exciting justification regarding the necessity of the MEP-pathway existence, not only for the production of the isoprenoids but also for retrograde signaling function of each of intermediates essential for coordinated action of the two organelles. This possibility could also explain the coexistence of MVA- and MEP-, the two isoprenoid producing pathways in plants.

### MEP-pathway genes are resistant to duplication

In land plants, all the MEP-pathway genes with the exception of *DXS*, are present as single-copy in all the analyzed diploid plants in spite of ancient WGD events. In fact, although *DXS* experienced duplications, only one copy maintained the MEP-pathway-based enzyme activity [19–21]. The critical nature of gene duplication as a source of evolutionary innovation and adaptation [34], raises the question of why the MEP-pathway genes have remained single-copies. One explanation might be that under the relaxation of selective pressure, the duplicated copy is inclined to accumulate deleterious mutations [35], which in turn could result in a dominant negative inhibition of the other functional copy. Indeed this is in stark contrast with the existence of multiple copies of the cytosolic MVA-pathway genes, such as functionally redundant *AACT1* and *AACT2* (anthocyanin-5-aromatic acyl transferase-like) both of which encode the initial enzyme of the MVA-pathway [36], or *HMG1* and *HMG2* encoding the HMGR (3-hydroxy-3-methylglutaryl CoA reductase) [37], and *MVD1* and *MVD2* encoding the MVD (mevalonate diphosphate decarboxylase) [38]. Plants lacking *AACT1* or *HMG2* are viable with no apparent phenotypes, in contrast to indispensability of MEP-pathway genes.

### The polyphyletic origin of MEP-pathway genes

Among seven MEP-pathway genes, *DXS* and *MDS* have originated from ‘other-eubacteria’. The closest sister clade of plastid-bearing eukaryotes DXS is alpha-proteobacteria, also the known ancestor of mitochondrion [39]. This suggests that plastid-bearing eukaryotes *DXS* might have originated directly from alpha-proteobacteria via horizontal gene transfer (HGT), or indirectly via endosymbiotic gene transfer (EGT) from the mitochondrion genome.

We were unable to place the origin of eukaryotic MDS, but through expanded phylogenetic analyses we determined notable homology between Chlamydiae and eukaryotes sequences of three (*CMS*, *CMK*, and *HDS*) of the seven MEP-pathway genes. Specifically, the phylogenetic trees of CMS and CMK, show that eukaryotes lineage form a sister cluster with the corresponding Chlamydiae gene, suggestive of HGTs between Chlamydiae and the common ancestor of eukaryotes. In addition, phylogenetic analyses of HDS depict clustering of red algae with cyanobacteria as opposed to other plastid-bearing eukaryotes that form a sister group with Chlamydiae. One potential explanation for this bifurcated clustering is that the ancestral plastid-bearing eukaryotes acquired *HDS* from Chlamydiae, but in red algae ancestor, the chlamydial *HDS* was lost as the result of two major phases of genome reduction [40], but later it was replaced by the second HGT event from cyanobacteria.

The necessity of Chlamydiae like HDS enzyme in plastid bearing organisms potentially could be justified as a response to the changing environmental conditions over time. Based on the oldest eukaryotic algae fossils findings in conjunction with the molecular clock data, plastids are predicted to have originated in Mesoproterozoic era ~ 0.9–1.7 billion years ago [41]. During the Proterozoic era, oxygen began to rise and built up to above 10% of the levels existed in the atmosphere at Mesoproterozoic era [42, 43]. Simultaneously, the earth entered a warm period ending glaciations and raising the tropical mean sea surface temperatures from ~ 19.4–28.7 °C [44].

It is well established that HDS, a [4Fe-4S]-protein reactive to oxygen species, is hypersensitive to high radiation and supra-optimal temperatures. Under these unfavorable conditions inhibition of HDS results in accumulation of its substrate, MEcPP, that in turn protects MEP-pathway activity by restricting oxidative stress [45–47]. Plastid-bearing eukaryotes are frequently and simultaneously exposed to reactive oxygen species, high light irradiance and hot temperatures, and one could consider HDS enzyme as the gatekeeper maintaining the MEP-pathway’s functionality.

Accordingly, we propose that the evolutionary pressures resulted from high oxygen and higher temperatures at the era of plastid establishment may have led to acquisition of a monomeric Chlamydiae like HDS in plastid-bearing organisms. The presence of a middle domain in the monomeric enzyme would have provided a higher ratio of protein/ labile [Fe-S]-iron cluster, thereby a functionally more efficient enzyme than the dimeric form in a high oxygen and high temperature environment. As such, plastid-bearing eukaryotes, acquired the more efficient monomeric HDS donated by Chlamydiae.

**Fig. 7:**
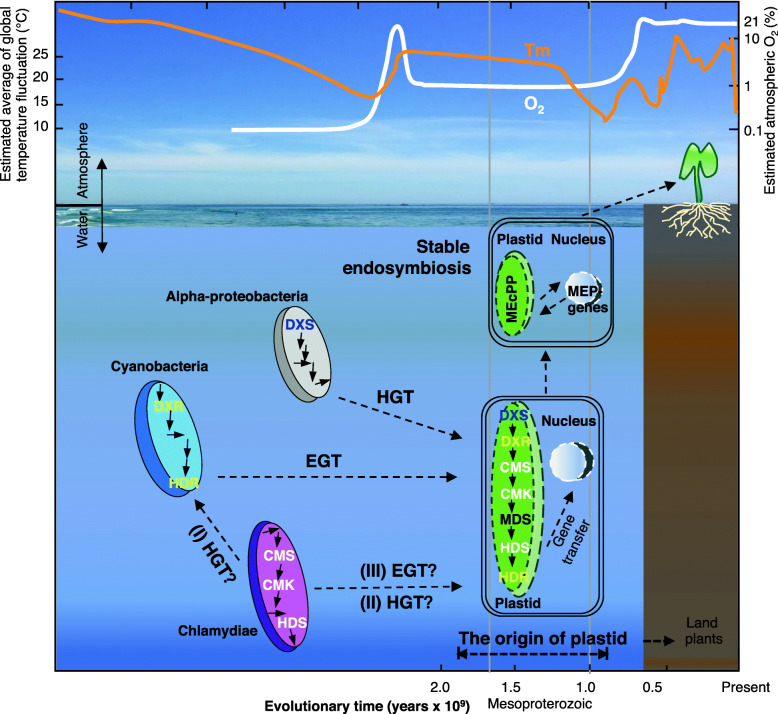
Schematic representation of the probable basis of polyphyletic origin of the MEP-pathway genes. The three proposed scenarios supporting polyphyletic origin of the MEP-pathway genes are: (I) Horizontal Gene Transfer (HGT) of the MEP-pathway genes from chlamydial (magenta) to cyanobacteria (cyan) pangenome followed by the cyanobacterial Endosymbiotic Gene Transfer (EGT) to the eukaryotic host; (II) HGT of genes from Chlamydiae-to-ancestral eukaryotes; (III) Co-contribution of cyanobacteria and Chlamydiae to the MEP-pathway genes. In all the probable cases, the events were ensued by the transfer of genetic material from the endosymbiont(s) to the host nuclear genome (white circle). The estimated average of global temperature (Tm) fluctuation and atmospheric oxygen (O_2_) are modified based on Harada, et al., 2015, Fiorella and Sheldon 2017, and Zhang et al., 2018. The authors depict the image by themselves

Our overall finding poses the question of how multiple donors could have contributed to the MEP-pathway. The simplified schematic (Fig. 7) depicts the three potential scenarios addressing the question. Scenario I proposes inherited chimerism by EGT, and that the occurrence of prokaryotic HGT to the cyanobacteria genome happened prior to endosymbiosis event leading to plastid formation [48]. If so, cyanobacteria must have acquired chlamydial MEP-pathway genes through HGT before EGT in plants (Fig. 7). In such a case, one would expect the presence of chlamydial type *CMS*, *CMK* and *HDS* sequences in *Gloeomargarita lithophora* genome, the prime candidate for extant relative of the cyanobacterial plastid progenitor [49]. However, clustering of the three genes in *G. lithophora* with cyanobacteria and not with Chlamydiae (Figs. S3-S4, and S6), diminishes the probability of scenario I.

Scenario II suggests that *CMS*, *CMK* and *HDS* in eukaryotes are the result of HGT from Chlamydiae after the endosymbiosis (Fig. 7). But, the inability of Chlamydiae to infect current photosynthetic eukaryotes or plastid-containing organisms renders this scenario less plausible.

Scenario III supports co-contribution of cyanobacteria and Chlamydiae to the origin of the primary plastid (Fig. 7), once proposed as the `tripartite (*ménage-à-trois*- ‘household of three’) symbiotic relationship between the extant order Chlamydiales, a cyanobacterium, and an eukaryotic host for the establishment of the eukaryotes lineages [50–53]. The tripartite endosymbiosis supported by phylogenomic analyses of a considerable number of nuclear genes in eukaryotes related to chlamydial homologues, proposes that the chlamydial partner injected effector proteins into the ancestral eukaryotes as a strategy to manipulate host cell carbohydrate metabolism to the parasite’s advantage [50, 51, 54]. However, counter arguments question the correct evolutionary models of phylogenomic analyses, the high frequency of HGTs among prokaryotes and among prokaryote-to-eukaryote [55–58]. Our analyses based on the best-fitting evolutionary models for constructing phylogenetic trees of individual MEP-pathway genes support the chlamydial origination for three of seven MEP-pathway genes in plastid-bearing eukaryotes, even though the evolutionary pressure(s) that led to plastid-bearing eukaryotes harboring a chimera MEP-pathway remains an enigma.

Our data clearly presents contribution of both cyanobacteria and Chlamydiae to plastid-bearing eukaryotes MEP-pathway and by extension to the origin of the primary plastid.

## Conclusion

The MEP-pathway genes are highly conserved and are essential for the survival of plastid-bearing eukaryotes. The plastid-bearing eukaryotes MEP-pathway genes originated from both cyanobacteria and Chlamydiae indicating their co-contributions to the evolution of primary plastids. The nuclear-encoded plastid-destined MEP-pathway enzymes enable the host eukaryotes to control plastids in a stable endosymbiosis system, while in return MEcPP, the plastid-produced intermediate of the MEP-pathway, coordinates expression of selected nuclear stress-response genes and the corresponding physiological ramifications. These bilateral controls mediated by MEP-pathway may also shed light on the basis of the co-existence of cytosolic and plastidial isopreneoid biosynthesis pathways in eukaryotes.

In summary, these findings uncover the evolutionary history and characteristics of the plastidial isoprenoid biosynthesis-pathway genes and its implications in origin and evolution of primary plastid.

## Methods

### Identification the homologues of MEP-pathway

Plant genome sequences were downloaded from the Phytozome v12 (https://phytozome.jgi.doe.gov/pz/portal.html), *Amborella* Genome Database (http://amborella.huck.psu.edu/data), Spruce Genome Project (http://congenie.org/start) and JGI Genome portal (https://genome.jgi.doe.gov/). Algea genomes were downloaded from Phytozome and Greenhouse (https://greenhouse.lanl.gov/greenhouse/organisms/). Annotated genome sequences of *Chara braunii* [59], *Klebsormidium nites* [60] are downloaded. Genome sequences of selected eubacteria were downloaded from Ensembl (release 90) (ftp://ftp.ensembl.org/pub/).

The names and IDs of MEP-pathway genes in *A. thaliana* are *DXS* (1-deoxy-D-xylulose-5-phosphate synthase): *AT4G15560*; *DXR* (1-deoxy-D-xylulose 5-phosphate reductoisomerase): *AT5G62790*; *CMS* (4-Diphosphocytidyl-2C-methyl-D-erythritol synthase): *AT2G02500*; *CMK* (4-(cytidine 5′-diphospho)-2-C-methyl-D-erythritol kinase activity): *AT2G26930*; *MDS* (2C-methyl-d-erythritol 2,4-cyclodiphosphate synthase): *AT1G63970*; *HDS* (4-hydroxy-3-methylbut-2-enyl diphosphate synthase): *AT5G60600*; and *HDR* (4-hydroxy-3-methylbut-2-en-1-yl diphosphate reductase): *AT4G34350*. The protein domain information for each MEP-pathway gene in *A. thaliana* was obtained from Phytozome v12, which are 1-deoxy-D-xylulose-5-phosphate synthase as PF13292 for DXS, 1-deoxy-D-xylulose-5-phosphate reductoisomerase as PF02670 and 1-deoxy-D-xylulose-5-phosphate reductoisomerase C-terminal as PF08436 for DXR, MobA-like NTP transferase as PF12804 for CMS, GHMP kinases N terminal as PF00288 and GHMP kinases C terminal as PF08544 for CMK, YgbB as PF02542 for MDS, GcpE as PF04551 for HDS, and LytB protein as PF02401 for HDR, respectively. Hidden Markov Models (HMM) [61] matrix presenting each domain of MEP-pathway enzymes was downloaded from Pfam (https://pfam.xfam.org/). Then, hmmsearch and fastacmd were used to obtain protein sequences in selected whole-genome sequenced species. And protein sequences of MEP-pathway genes in PVC bacteria were retrieved from PVCbase (http://pvcbacteria.org/pvcbase/) using BLASTP. All the identified proteins were examined on Pfam website to confirm the presence of the corresponding protein domain.

### Multiple sequence alignment and phylogenetic tree construction

The MEP-pathway protein sequences were aligned using MUSCLE [62] v3.8.31 with default parameters. Prottest [63] was used to select out the best-fitting evolutionary model for each aligned protein matrix of MEP-pathway gene. Then the evolutionary model of WAG + G was specified for DXS, LG + I + G was specified for DXR, HDS and HDR, and VT + I + G was specified for CMS, CMK and MDS. Phylogenetic trees were constructed by RAxML [64] v7.1.0 and MrBayes [65] v3.2.7. As an exception to the MEP-pathway genes, the *CMK* belongs to the GHMP gene family with 13 copies in *A. thaliana*. All protein sequences of this family in each species were firstly retrieved and preliminary ML tree using aligned sequences was constructed. Lastly, members of CMK and MVK (mevalonate kinase in the MVA-pathway) were selected out for constructing the final phylogenetic tree. The MVK branch was set as outgroup.

### Synteny analysis

The Locus search function in PGDD [66] (http://chibba.agtec.uga.edu/duplication/), a public database for cluster identification of plant genes based on intra- or cross-genome syntenic relationships, was implemented for identifying the intra-species duplication blocks around 500 kb region of each MEP-pathway genes in *A. thaliana* and *O. sativa.*

### dN/dS analyses

Nucleotide sequences for represented species in each lineage, namely *A. thaliana* a eudicot, *O. sativa* a monocot, *A. trichopoda* an early-diverging angiosperm, *P. abies* a gymnosperm, *P. patens* a moss, *S. moellendorffii* a lycophyte, *Volvox carteri* a green algae and *Cyanidioschyzon merolae* a red algae, were retrieved to calculate the nonsynonymous to synonymous rate ratio (ω = dN/dS) between *A. thaliana* and all other species. The ω was calculated by yn00 contained in the software PAML v4.5 [67] using the Yang & Nielsen method, wehre 0 < ω < 1 indicates purifying selection, ω = 1 corresponds to neutral selection, and ω > 1 implicates positive selection. The distributions of all dN/dS values for each MEP-pathway gene were drawn by the boxplot function in the R [68] program.

### Expression levels, codon adaption index (CAI)

The sources of the RPKM values of all expressed genes in wild type plant in represented species were listed as following: *A. thaliana* [69], *Z. mays* [70] (2 replicates of mock treated wild type), *G. max* (SoyBase Soybean Genome Annotation Page: https://soybase.org/soyseq/tables_lists/index.php), *O. sativa* (Rice Genome Annotation Project: http://rice.plantbiology.msu.edu/expression.shtml, use four libraries of four-leaf stage seedling), *P. abies* (Spruce Genome Project: ftp://plantgenie.org/Data/ConGenIE/Picea_abies/v1.0/Expression/), *P. patens* [71] and *S. moellendorffii* [72]. Genes with RPKM ≥1 were retained for further analyses. The relative expression ranking of each gene was calculated using the formula: 1- (order of the gene) / (total number of all expressed genes). The relative expression ranking of all represented species for each gene were presented as scatter-boxplot in the R program.

Nucleotide sequences for each MEP-pathway gene in selected species were retrieved in corresponding datasets. Codon usage table for each selected species was obtained from Condon Usage Database (http://www.kazusa.or.jp/codon/). Lastly, fasta format of each nucleotide sequence and codon usage table of each species were inputted to calculate the CAI on the CAIcal SERVER [73] (http://ppuigbo.me/programs/CAIcal/).

### Plant material and growth conditions

We employed the RNAi lines for all MEP-pathway genes in *A. thaliana* that were previously generated [15]. Sterilized seeds sowed on 1/2 MS medium were maintained for 48 h at 4 °C. Two-week-old seedlings were grown at 22 °C under 16/8 h light/dark period.

## Supplementary Information

**Additional file 1: Figure S1.** Phylogenetic tree of DXS. **Figure S2.** Phylogenetic tree of MDS. **Figure S3.** Phylogenetic tree of DXR. **Figure S4.** Phylogenetic tree of HDR. **Figure S5.** Phylogenetic tree of CMS. **Figure S6.** Phylogenetic tree of CMK. **Figure S7.** Phylogenetic tree of HDS.

**Additional file 2: Table S1.** Synonymous (dN) and nonsynonymous (dS) substitution rates estimated by PAML. **Table S2.** The relative expression ratio of the MEP-pathway genes in represented species. **Table S3.** The Codon Adaption Index of the MEP-pathway genes.

**Additional file 3: Data S1.** Paralogous syntenic blocks display loss of duplicated copies of MEP-pathway genes.

## Data Availability

The datasets of phylogenetic matrices analyzed in the study are available in the figshare: https://figshare.com/articles/dataset/DXS_fas/13714165, https://figshare.com/articles/dataset/DXR_fas/13714174, https://figshare.com/articles/dataset/CMS_fas/13714180, https://figshare.com/articles/dataset/CMK_fas/13714207, https://figshare.com/articles/dataset/MDS_fas/13714168, https://figshare.com/articles/dataset/HDS_fas/13714177, https://figshare.com/articles/dataset/HDR_fas/13714171.

## Abbreviations

MVA: Mevalonate
MEP: Methylerythritol phosphate
WGD: Whole genome duplication
PVC: Planctomycetes, Verrucomicrobia and Chlamydiae
dN: Non-synonymous substitution
dS: Synonymous substitution
MEcPP: Methylerythritol cyclodiphosphate
HGT: Horizontal gene transfer
