# Supplementary material for: The eukaryotic MEP-pathway genes are evolutionarily conserved and originated from Chlaymidia and cyanobacteria
Source: BMC Genomics. 2021 Feb 26;22:137. doi: 10.1186/s12864-021-07448-x (PMC7912892; doi:10.1186/s12864-021-07448-x)
Supplement: Supplementary file 1 — Additional file 1: Figure S1. Phylogenetic tree of DXS. Figure S2. Phylogenetic tree of MDS. Figure S3. Phylogenetic tree of DXR. Figure S4. Phylogenetic tree of HDR. Figure S5. Phylogenetic tree of CMS. Figure S6. Phylogenetic tree of CMK. Figure S7. Phylogenetic tree of HDS. [file 12864_2021_7448_MOESM1_ESM.pdf]

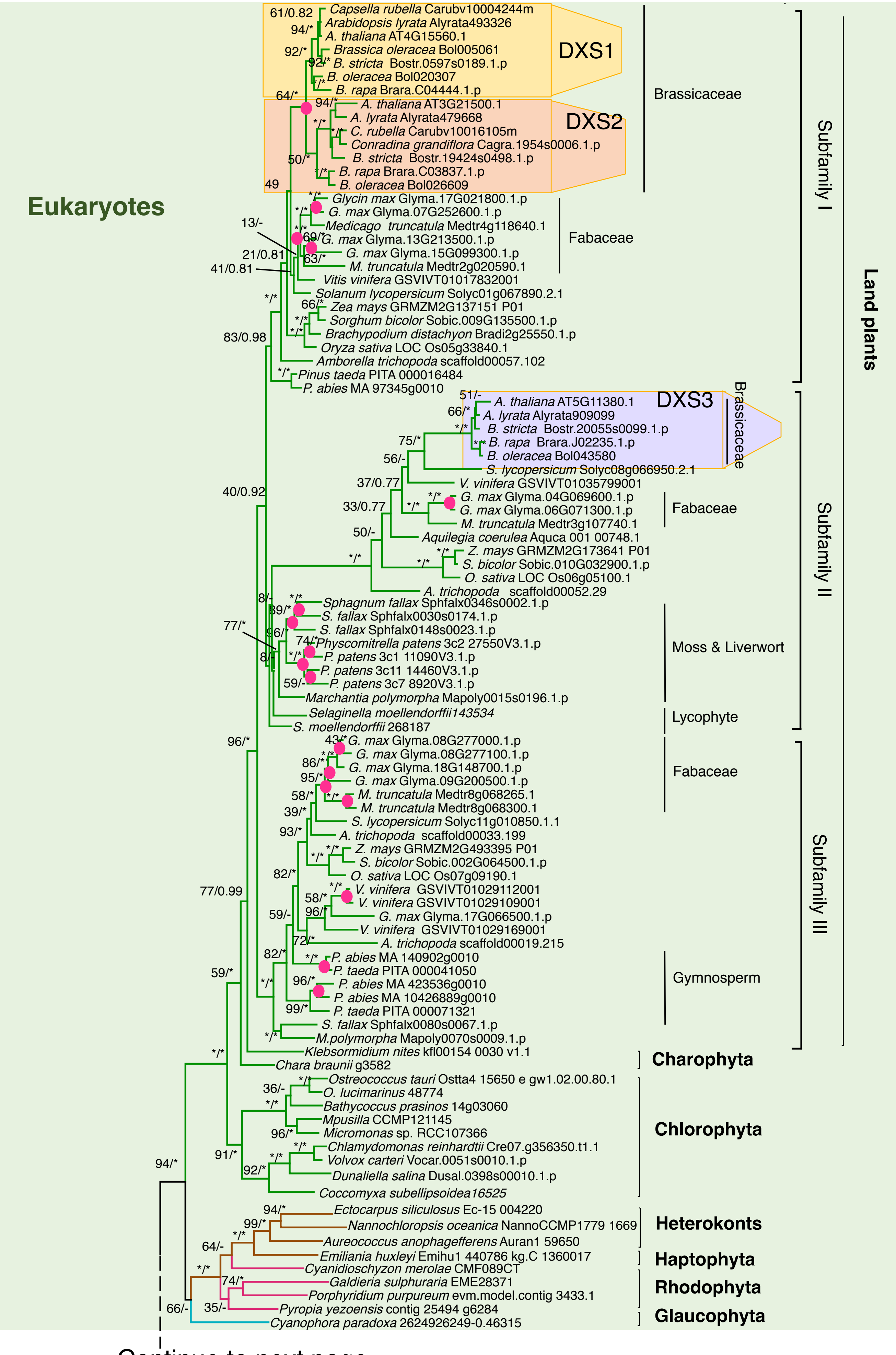

Continue to next page

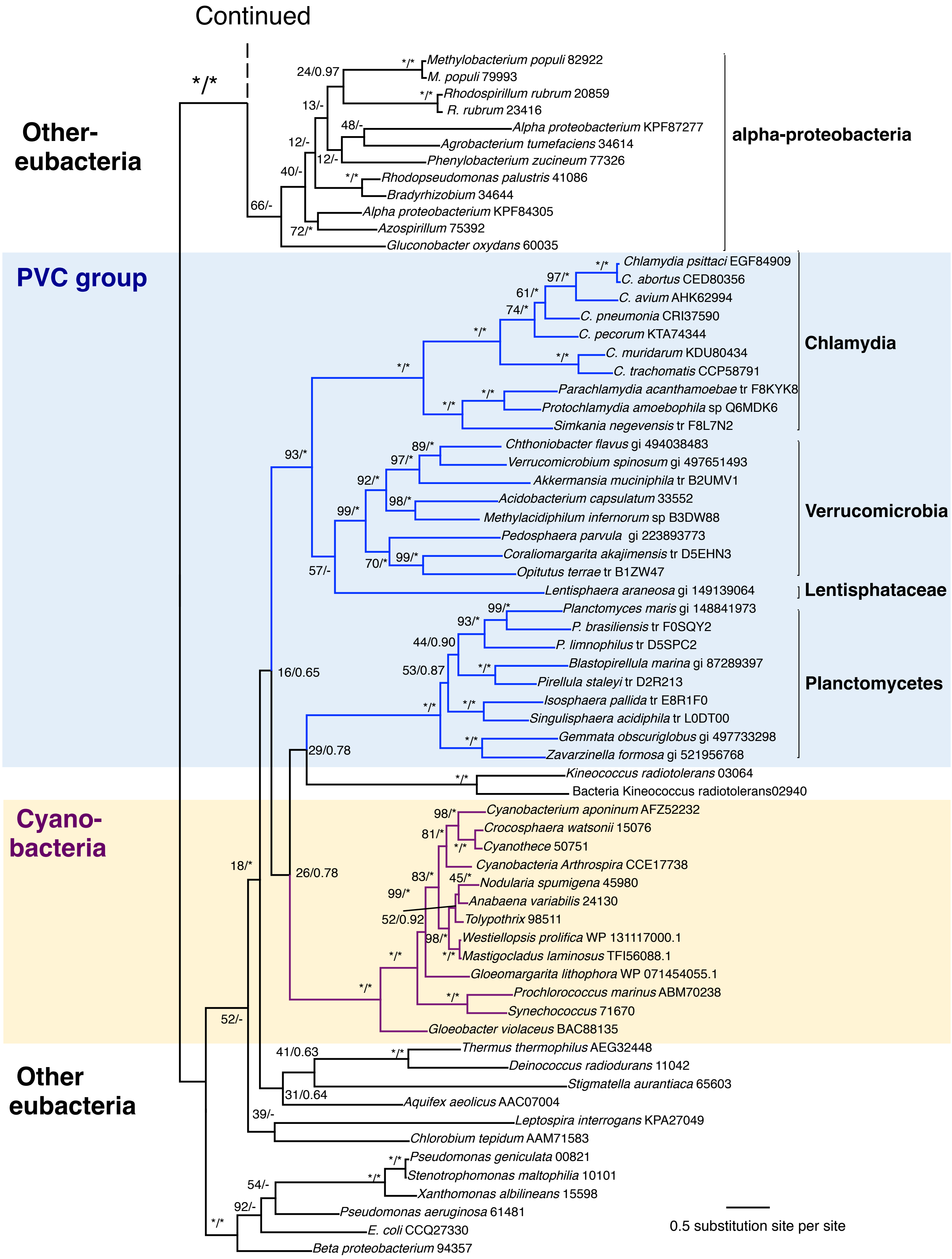

Figure S1. Phylogenetic tree of DXS. Numbers associated with branches are bootstrap values (BS, on the left) obtained by RAxML and the posterior probability (PP, on the right) obtain by MrBayes. Taxa in different major groups are shaded in different colors. The star is the BS=100 or PP=1.0. Pink-filled circle indicates gene duplication. Dash means the topology is different between two methods.

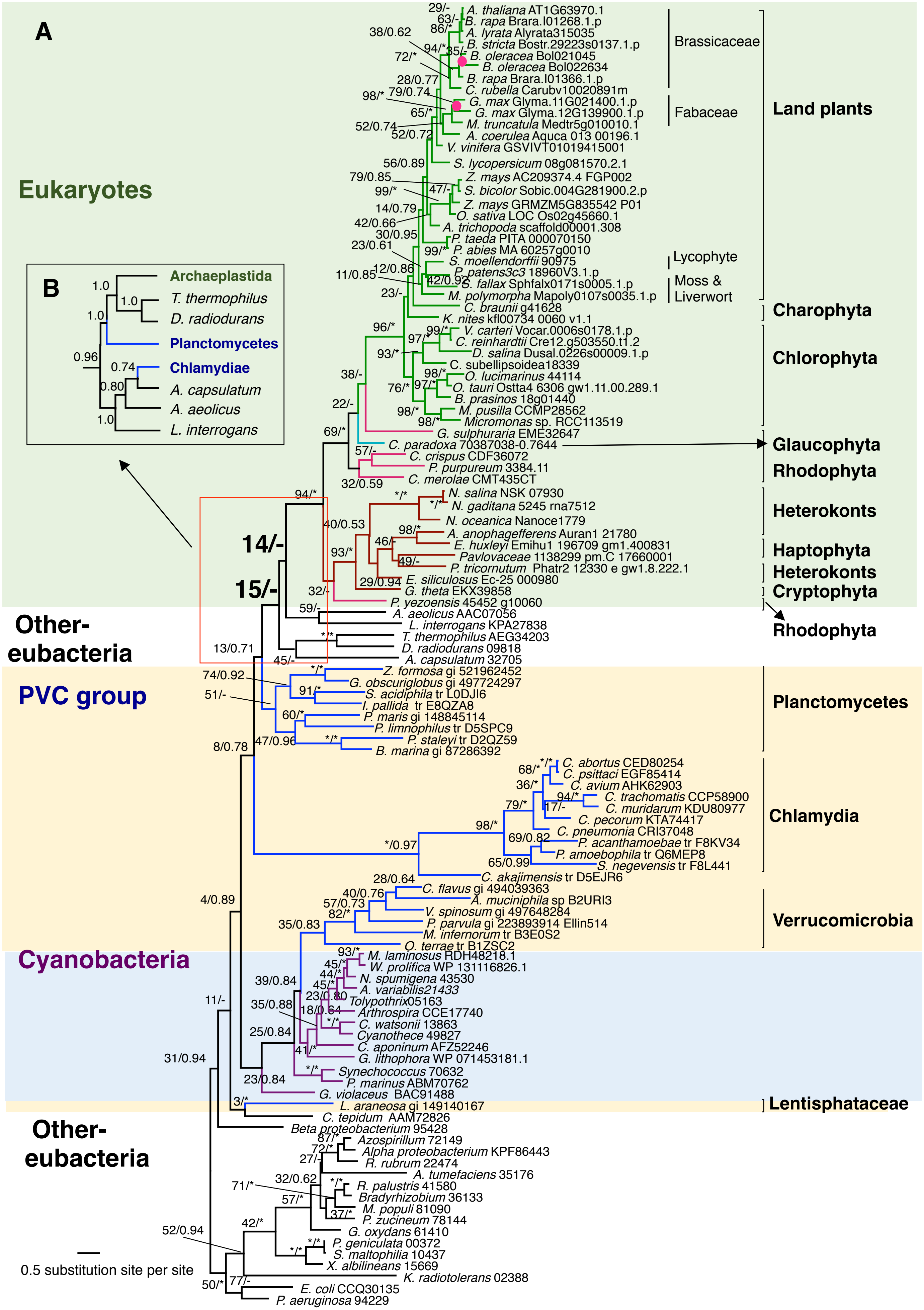

Figure S2. Phylogenetic tree of MDS. A.The detailed phylogenetic tree of RAxML and MrBayes. **B**. The simplified cladogram shows the different eubacteria species clustered with eukaryotes obtained from MrBayes comparing to RAxML shown in red rectange. Numbers associated with branches are BS values values (on the left) obtained by RAxML and the PP values (on the right) obtain by MrBayes. Taxa in different major groups are shaded in different colors. The star is the BS=100 or PP=1.0. Pink-filled circle indicates gene duplication. Dash means the topology is different between two methods.

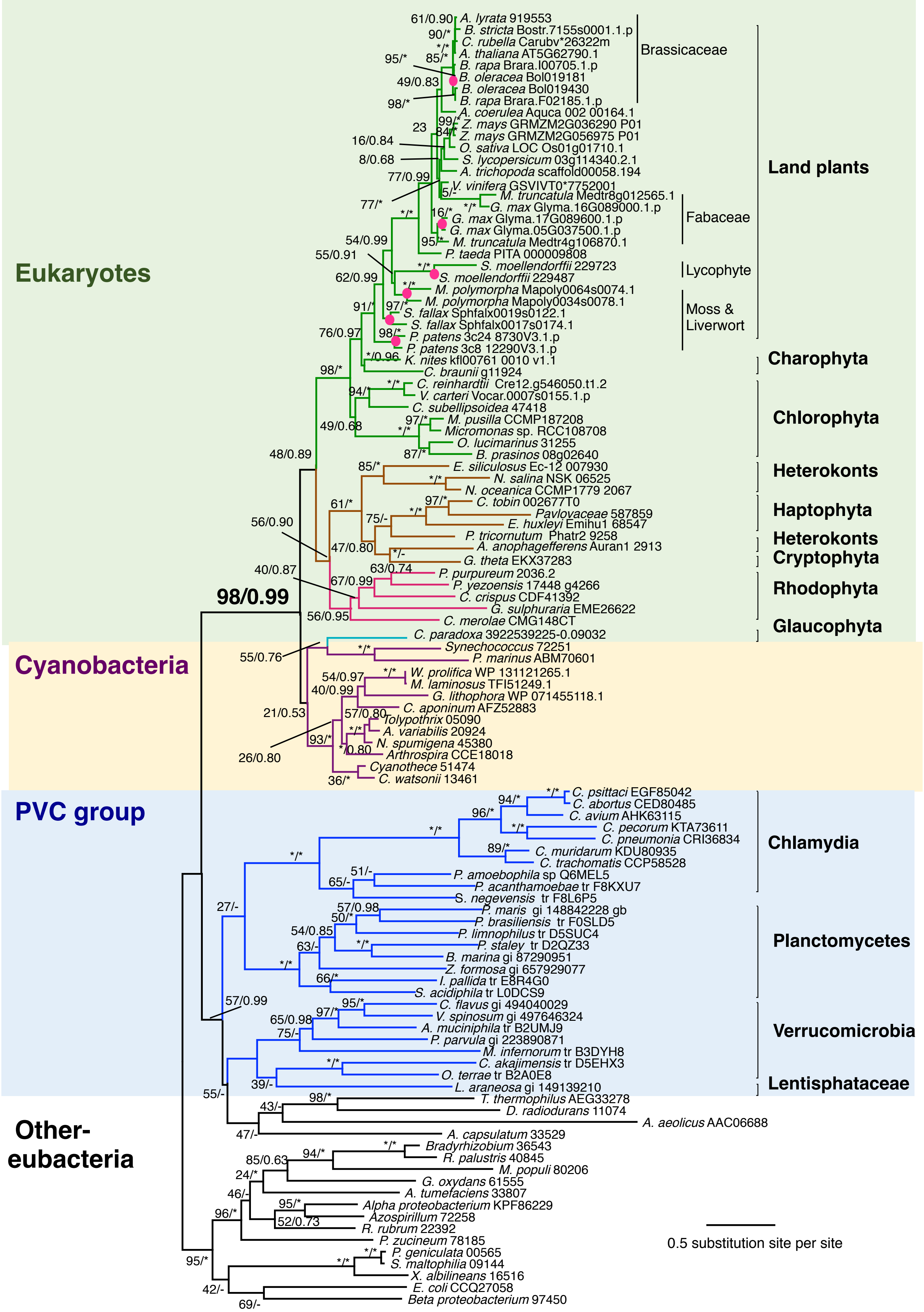

Figure S3. Phylogenetic tree of DXR. Numbers associated with branches are BS values (on the left) obtained by RAxML and the PP values (on the right) obtain by MrBayes. Taxa in different major groups are shaded in different colors. The star is the BS=100 or PP=1.0. Pink-filled circle indicates gene duplication. Dash means the topology is different between two methods.

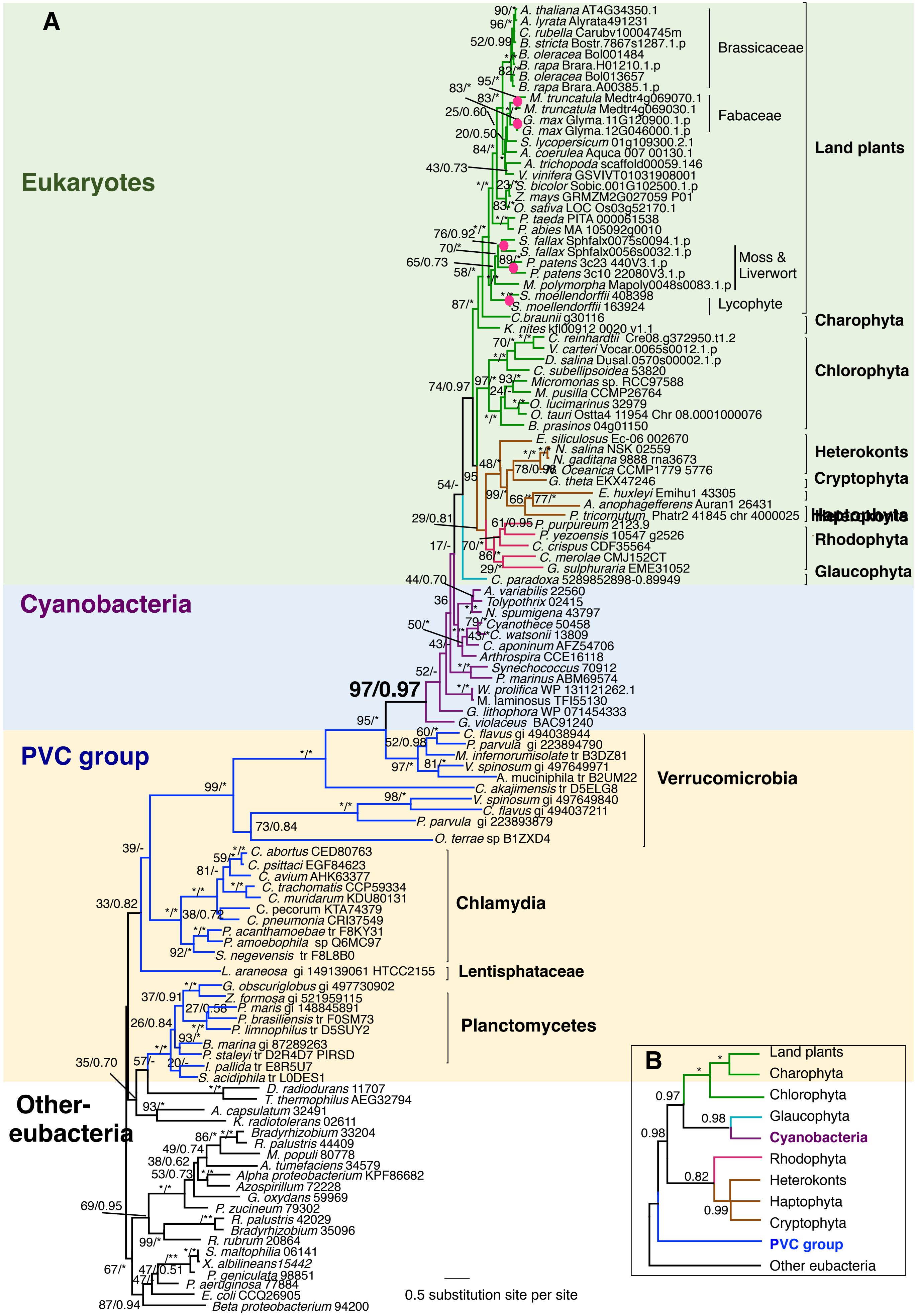

Figure S4. Phylogenetic tree of HDR. A. The detailed phylogenetic tree of RAxML and MrBayes. **B**. he simplified cladogram shows the different position of Cyanobacteria obtained from MrBayes. Numbers associated with branches are BS values values (on the left) obtained by RAxML and the PP values (on the right) obtain by MrBayes. Taxa in different major groups are shaded in different colors. The star is the BS=100 or PP=1.0. Pink-filled circle indicates gene duplication. Dash means the topology is different between two methods.

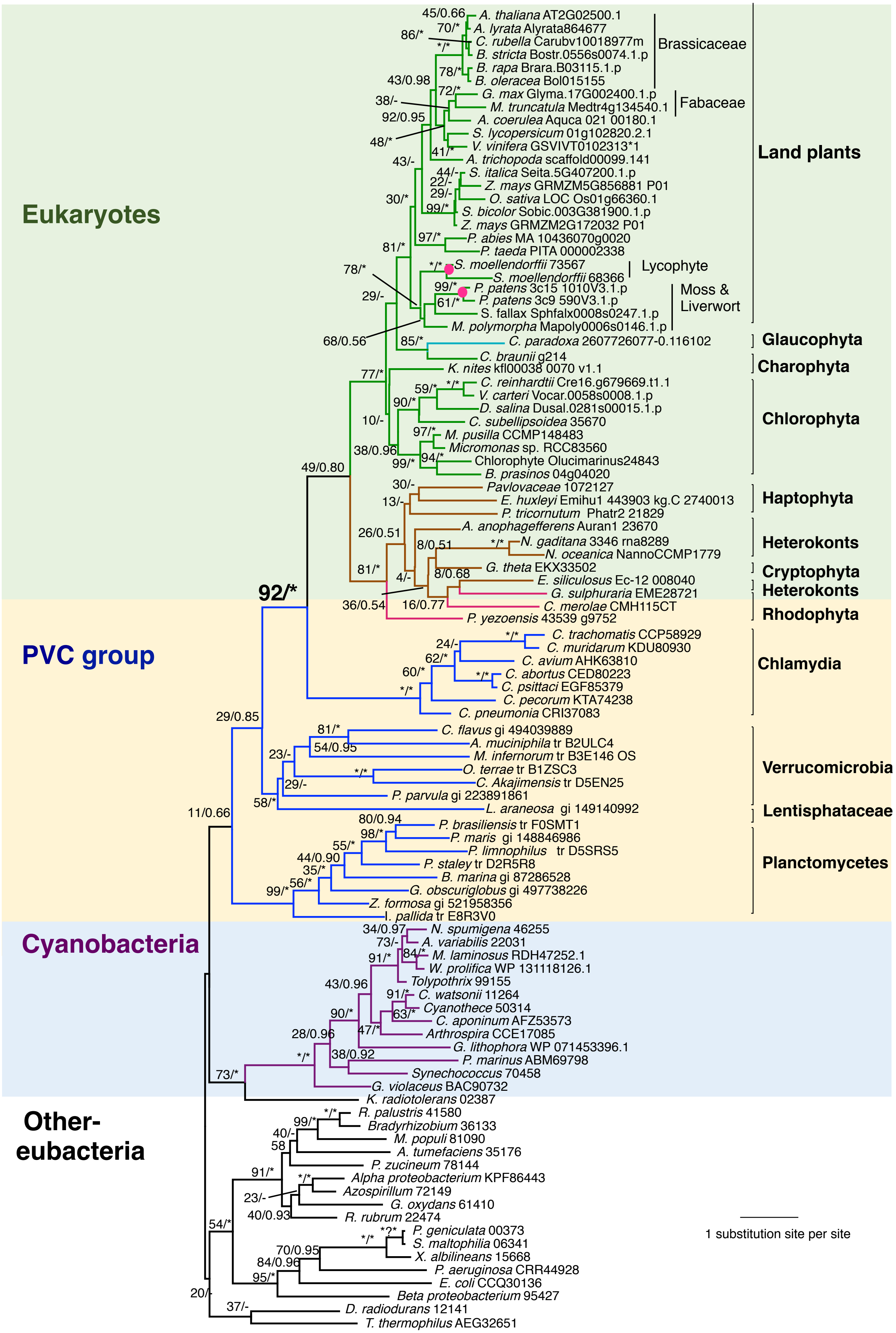

Figure S5. Phylogenetic tree of CMS. Numbers associated with branches are BS values values (on the left) obtained by RAxML and the PP values (on the right) obtain by MrBayes. Taxa in different major groups are shaded in different colors. The star is the BS=100 or PP=1.0. Dash means the topology is different between two methods. Dash means the topology is different between two methods.

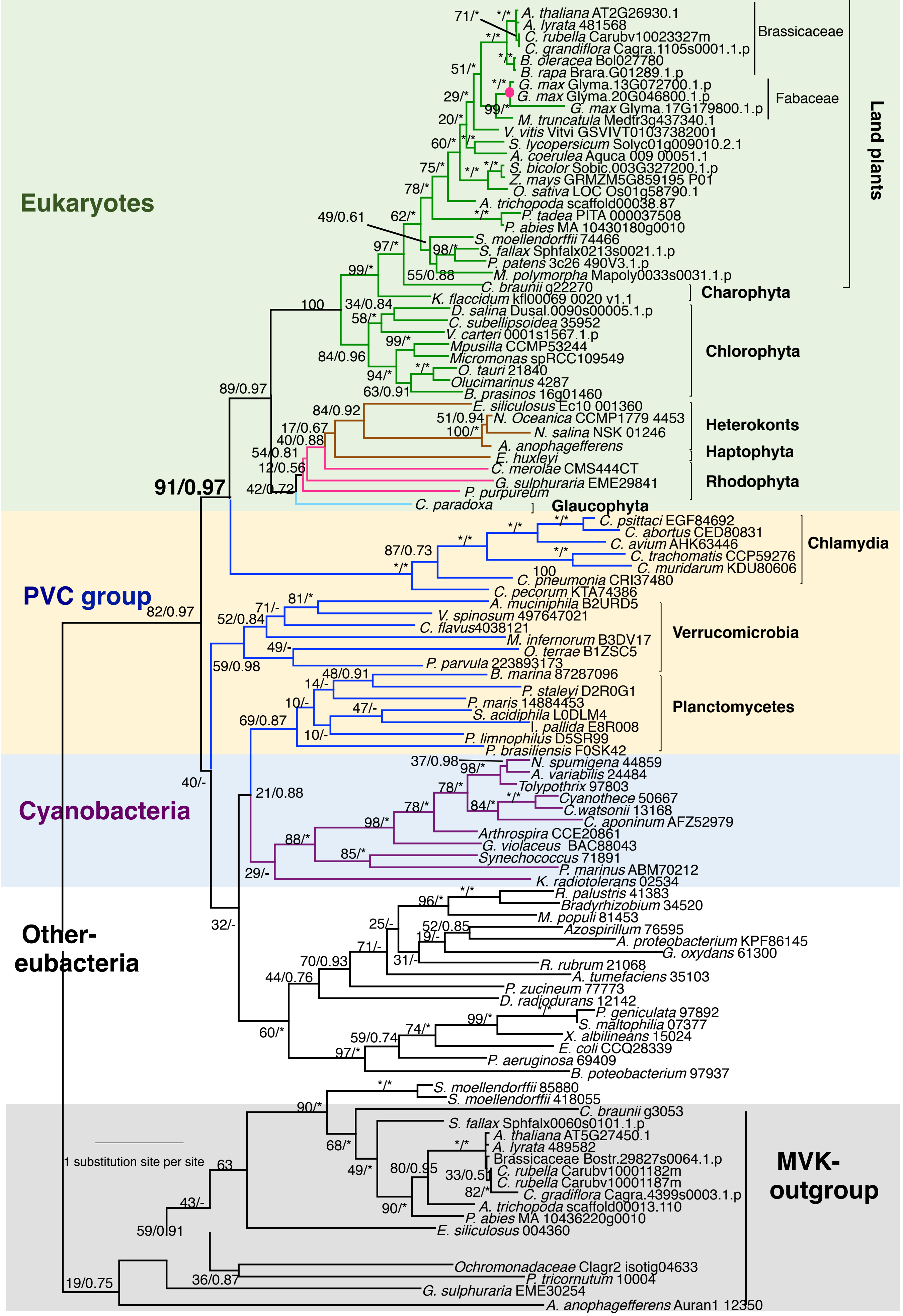

Figure S6. Phylogenetic tree of CMK. MVK sequences are set as the outgroup. Numbers associated with branches are BS values values (on the left) obtained by RAxML and the PP values (on the right) obtain by MrBayes. Taxa in different major groups are shaded in different colors. The star is the BS=100 or PP=1.0. Dash means the topology is different between two methods.

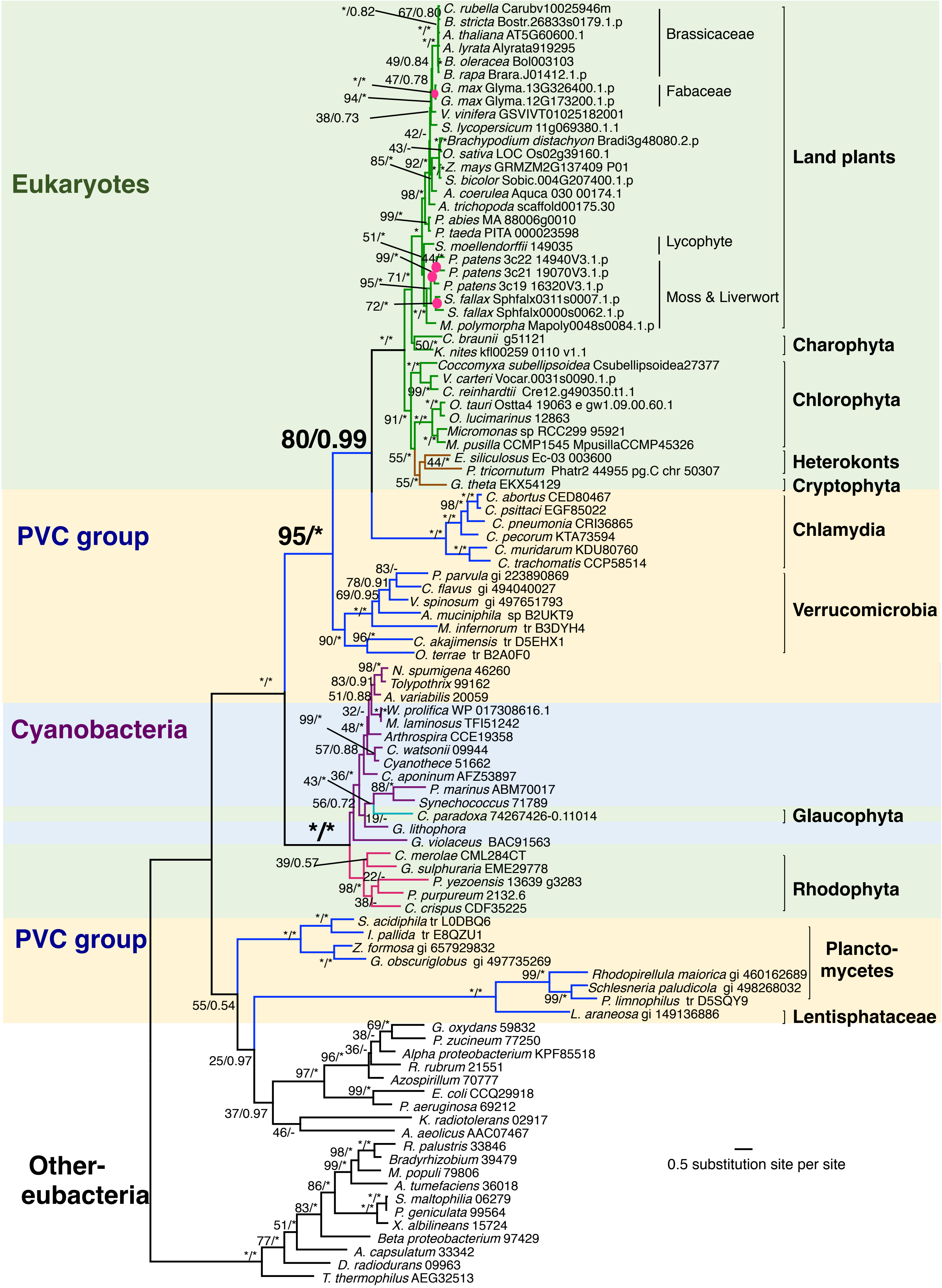

Figure S7. Phylogenetic tree of HDS. Numbers associated with branches are BS values values (on the left) obtained by RAxML and the PP values (on the right) obtain by MrBayes. Taxa in different major groups are shaded in different colors. The star is the BS=100 or PP=1.0. The dash is the topology is different obtained by MrBayes. Pink-filled circle indicates gene duplication. Dash means the topology is different between two methods.
