# Supplementary material for: The eukaryotic MEP-pathway genes are evolutionarily conserved and originated from Chlaymidia and cyanobacteria
Source: BMC Genomics. 2021 Feb 26;22:137. doi: 10.1186/s12864-021-07448-x (PMC7912892; doi:10.1186/s12864-021-07448-x)
Supplement: Supplementary file 2 — Additional file 2: Table S1. Synonymous (dN) and nonsynonymous (dS) substitution rates estimated by PAML. Table S2. The relative expression ratio of the MEP-pathway genes in represented species. Table S3. The Codon Adaption Index of the MEP-pathway genes. [file 12864_2021_7448_MOESM2_ESM.docx]

**Table S1.** Synonymous (dN) and nonsynonymous (dS) substitution rates estimated by PAML.

| **Gene** | **Species 1** | **Species 2** | **Lineages for the species 2** | **dN** | **dS** | **dN/dS** |
| --- | --- | --- | --- | --- | --- | --- |
| ***DXS*** | *A. thaliana* | *Oryza sativa* | Monocots in flowering plant | 0.1343 | 4.7214 | 0.0284 |
|  |  | *Amborella trichopoda* | Basal flowering plant | 0.1631 | 4.7489 | 0.0343 |
|  |  | *Picea abies* | Gymnosperms | 0.171 | 4.6628 | 0.0367 |
|  |  | *Selaginella moellendorffii* | Lycophytes | 0.1293 | 4.6448 | 0.0278 |
|  |  | *P. patens* | Moss | 0.2047 | 4.7331 | 0.0432 |
|  |  | *Volvox carteri* | Green algae | 0.3244 | 4.7389 | 0.0685 |
|  |  | *Cyanidioschyzon merolae* | Red algae | 0.3749 | 4.4286 | 0.0847 |
|  |  | *Cyanobacterium aponinum* | Cyanobacteria | 0.5194 | 4.5019 | 0.1154 |
|  |  | *Escherichia coli* | Non-cyanobacteria | 0.5492 | 4.6256 | 0.1187 |
| ***DXR*** | *A. thaliana* | *O. sativa* | Monocots | 0.124 | 2.1508 | 0.0577 |
|  |  | *A. trichopoda* | Basal flowering plant | 0.1282 | 2.191 | 0.0585 |
|  |  | *P. abies* | Gymnosperms | 0.6294 | 2.5698 | 0.2449 |
|  |  | *S. moellendorffii* | Lycophytes | 0.2457 | 2.6487 | 0.0928 |
|  |  | *P. apatens* | Moss | 0.238 | 4.3873 | 0.0542 |
|  |  | *V. carteri* | Green algae | 0.2935 | 4.3493 | 0.0675 |
|  |  | *C. merolae* | Red algae | 0.4442 | 4.3994 | 0.1010 |
|  |  | *C. aponinum* | Cyanobacteria | 0.3108 | 4.1553 | 0.0748 |
|  |  | *E. coli* | Non-cyanobacteria | 0.6135 | 4.2484 | 0.1444 |
| ***CMS*** | *A. thaliana* | *O. sativa* | Monocots | 0.372 | 2.1463 | 0.1733 |
|  |  | *A. trichopoda* | Basal flowering plant | 0.2619 | 2.1961 | 0.1193 |
|  |  | *P. abies* | Gymnosperms | 0.7975 | 3.4802 | 0.2292 |
|  |  | *S. moellendorffii* | Lycophytes | 0.2948 | 2.1002 | 0.1404 |
|  |  | *P. apatens* | Moss | 0.4162 | 4.0716 | 0.1022 |
|  |  | *V. carteri* | Green algae | 0.4473 | 3.9586 | 0.1130 |
|  |  | *C. merolae* | Red algae | 0.7782 | 2.3801 | 0.3270 |
|  |  | *C. aponinum* | Cyanobacteria | 0.7554 | 3.7226 | 0.2029 |
|  |  | *E. coli* | Non-cyanobacteria | 0.8275 | 2.2901 | 0.3613 |
| ***CMK*** | *A. thaliana* | *O. sativa* | Monocots | 0.2624 | 2.0172 | 0.1301 |
|  |  | *A. trichopoda* | Basal flowering plant | 0.2365 | 4.1375 | 0.0572 |
|  |  | *P. abies* | Gymnosperms | 0.3171 | 4.2034 | 0.0754 |
|  |  | *S. moellendorffii* | Lycophytes | 0.2931 | 2.6135 | 0.1121 |
|  |  | *P. apatens* | Moss | 0.3581 | 3.0147 | 0.1188 |
|  |  | *V. carteri* | Green algae | 0.5568 | 4.1624 | 0.1338 |
|  |  | *C. merolae* | Red algae | 0.6486 | 4.2391 | 0.1530 |
|  |  | *E. coli* | Non-cyanobacteria | 0.8250 | 3.9772 | 0.2074 |
| ***MDS*** | *A. thaliana* | *O. sativa* | Monocots | 0.2691 | 3.9021 | 0.0690 |
|  |  | *A. trichopoda* | Basal flowering plant | 0.1402 | 3.6094 | 0.0388 |
|  |  | *P. abies* | Gymnosperms | 0.7542 | 3.4331 | 0.2197 |
|  |  | *S. moellendorffii* | Lycophytes | 0.2683 | 3.9602 | 0.0677 |
|  |  | *P. apatens* | Moss | 0.3534 | 3.8818 | 0.0910 |
|  |  | *V. carteri* | Green algae | 0.3418 | 3.7254 | 0.0917 |
|  |  | *C. merolae* | Red algae | 0.4469 | 3.846 | 0.1162 |
|  |  | *C. aponinum* | Cyanobacteria | 0.5691 | 3.4151 | 0.1666 |
|  |  | *E. coli* | Non-cyanobacteria | 0.533 | 3.6611 | 0.1456 |
| ***HDS*** | *A. thaliana* | *O. sativa* | Monocots | 0.1295 | 1.9391 | 0.0668 |
|  |  | *A. trichopoda* | Basal flowering plant | 0.1151 | 2.0216 | 0.0569 |
|  |  | *P. abies* | Gymnosperms | 0.1379 | 4.6845 | 0.0294 |
|  |  | *S. moellendorffii* | Lycophytes | 0.174 | 4.6993 | 0.0370 |
|  |  | *P. apatens* | Moss | 0.1987 | 4.7182 | 0.0421 |
|  |  | *V. carteri* | Green algae | 0.3107 | 4.6311 | 0.0671 |
|  |  | *C. merolae* | Red algae | 0.5678 | 4.3383 | 0.1309 |
|  |  | *C. aponinum* | Cyanobacteria | 0.4541 | 4.2068 | 0.1079 |
|  |  | *E. coli* | Non-cyanobacteria | 0.6713 | 4.2057 | 0.1596 |
| ***HDR*** | *A. thaliana* | *O. sativa* | Monocots | 0.1758 | 4.3057 | 0.0408 |
|  |  | *A. trichopoda* | Basal flowering plant | 0.1827 | 2.6296 | 0.0695 |
|  |  | *P. abies* | Gymnosperms | 0.2129 | 2.6724 | 0.0797 |
|  |  | *S. moellendorffii* | Lycophytes | 0.2339 | 2.1972 | 0.1065 |
|  |  | *P. apatens* | Moss | 0.2701 | 4.3323 | 0.0623 |
|  |  | *V. carteri* | Green algae | 0.4298 | 4.3021 | 0.0999 |
|  |  | *C. merolae* | Red algae | 0.4531 | 4.2863 | 0.1057 |
|  |  | *C. aponinum* | Cyanobacteria | 0.3244 | 4.1604 | 0.0780 |
|  |  | *E. coli* | Non-cyanobacteria | 0.8318 | 4.0392 | 0.2059 |

**Table S2.** The relative expression ratio of MEP pathway genes in represented species.

| **Life lineage** | **Species** | **Gene** | **ID** | **The expression rank in all expressed genes** | **Total number of genes (RPKM >=1)** | **The relative expression ratio** |
| --- | --- | --- | --- | --- | --- | --- |
| **Eudicot** | ***A. thaliana*** | *DXS* | AT4G15560 | 891 | 19175 | 95.35% |
|  |  | *DXR* | AT5G62790 | 2715 | 19175 | 85.84% |
|  |  | *CMS* | AT2G02500 | 6276 | 19175 | 67.27% |
|  |  | *CMK* | AT2G26930 | 8527 | 19175 | 55.53% |
|  |  | *MDS* | AT1G63970 | 6578 | 19175 | 65.69% |
|  |  | *HDS* | AT5G60600 | 1168 | 19175 | 93.91% |
|  |  | *HDR* | AT4G34350 | 919 | 19175 | 95.21% |
|  | ***G. max*** | *DXS* | Glyma.17G021800 | 5909 | 33853 | 82.55% |
|  |  | *DXS* | Glyma.07G252600 | 4256 | 33853 | 87.43% |
|  |  | *DXR* | Glyma.17G089600 | 17502 | 33853 | 48.30% |
|  |  | *DXR* | Glyma.05G037500 | 12015 | 33853 | 64.51% |
|  |  | *CMS* | Glyma.17G002400 | 6345 | 33853 | 81.26% |
|  |  | *CMK* | Glyma.17G179800 | 17501 | 33853 | 48.30% |
|  |  | *CMK* | Glyma.20G046800 | 23551 | 33853 | 30.43% |
|  |  | *CMK* | Glyma.13G072700 | 27659 | 33853 | 18.30% |
|  |  | *MDS* | Glyma.11G021400 | 4727 | 33853 | 86.04% |
|  |  | *MDS* | Glyma.12G139900 | 3010 | 33853 | 91.11% |
|  |  | *HDS* | Glyma.12G173200 | 7373 | 33853 | 78.22% |
|  |  | *HDS* | Glyma.13G326400 | 13491 | 33853 | 60.15% |
|  |  | *HDR* | Glyma.12G046000 | 2152 | 33853 | 93.64% |
|  |  | *HDR* | Glyma.11G120900 | 4960 | 33853 | 85.35% |
| **Monocot** | ***O. sativa*** | *DXS* | LOC_Os05g33840 | 474 | 19570 | 97.58% |
|  |  | *DXR* | LOC_Os01g01710 | 682 | 19570 | 96.52% |
|  |  | *CMS* | LOC_Os01g66360 | 4965 | 19570 | 74.63% |
|  |  | *CMK* | LOC_Os01g58790 | 5478 | 19570 | 72.01% |
|  |  | *MDS* | LOC_Os02g45660 | 328 | 19570 | 98.32% |
|  |  | *HDS* | LOC_Os02g39160 | 300 | 19570 | 98.47% |
|  |  | *HDR* | LOC_Os03g52170 | 215 | 19570 | 98.90% |
|  | ***Zea mays*** | *DXS* | GRMZM2G137151 | 11329 | 21641 | 47.65% |
|  |  | *DXS* | GRMZM2G173641 | 14554 | 21641 | 32.75% |
|  |  | *DXS* | GRMZM2G493395 | 18650 | 21641 | 13.82% |
|  |  | *CMS* | GRMZM2G172032 | 13448 | 21641 | 37.86% |
|  |  | *CMK* | GRMZM5G859195 | 3900 | 21641 | 81.98% |
|  |  | *MDS* | AC209374.4 | 5177 | 21641 | 76.08% |
|  |  | *MDS* | GRMZM5G835542 | 6646 | 21641 | 69.29% |
|  |  | *HDS* | GRMZM2G137409 | 4034 | 21641 | 81.36% |
|  |  | *HDR* | GRMZM2G027059 | 4786 | 21641 | 77.88% |
| **Gymnosperm** | ***P. abies*** | *CMS* | 10436070g0020 | 150464 | 549027 | 72.59% |
|  |  | *CMK* | 10430180g0010 | 15334 | 549027 | 97.21% |
|  |  | *HDR* | 105092g0010 | 1131 | 549027 | 99.79% |
| **Lycophyte** | ***S. moellendorffii*** | *DXS* | 268187 | 485 | 14669 | 96.69% |
|  |  | *DXS* | 143534 | 8342 | 14669 | 43.13% |
|  |  | *DXR* | 229487 | 1419 | 14669 | 90.33% |
|  |  | *CMS* | 73567 | 4845 | 14669 | 66.97% |
|  |  | *CMK* | 74466 | 7244 | 14669 | 50.62% |
|  |  | *MDS* | 90975 | 713 | 14669 | 95.14% |
|  |  | *HDS* | 149035 | 2235 | 14669 | 84.76% |
|  |  | *HDR* | 163924 | 958 | 14669 | 93.47% |
| **Moss** | ***P. patens*** | *DXS* | Pp3c2_27550V3.1 | 1473 | 16189 | 90.90% |
|  |  | *DXS* | Pp3c11_14460V3.1 | 15181 | 16189 | 6.23% |
|  |  | *DXS* | Pp3c1_11090V3.1 | Undetected | 16189 | NA |
|  |  | *DXS* | Pp3c7_8920V3.1 | Undetected | 16189 | NA |
|  |  | *DXR* | Pp3c8_12290V3.1 | 1453 | 16189 | 91.02% |
|  |  | *DXR* | Pp3c24_8730V3.1 | 2638 | 16189 | 83.70% |
|  |  | *CMS* | Pp3c9_590V3.1 | 8330 | 16189 | 48.55% |
|  |  | *CMS* | Pp3c15_1010V3.1 | 3217 | 16189 | 80.13% |
|  |  | *CMK* | Pp3c26_490V3.1 | 9289 | 16189 | 42.62% |
|  |  | *MDS* | Pp3c3_18960V3.1 | 2114 | 16189 | 86.94% |
|  |  | *HDS* | Pp3c19_16320V3.1 | 1365 | 16189 | 91.57% |
|  |  | *HDS* | Pp3c22_14940V3.1 | Undetected | 16189 | NA |
|  |  | *HDS* | Pp3c21_19070V3.1 | Undetected | 16189 | NA |
|  |  | *HDR* | Pp3c10_22080V3.1 | 2027 | 16189 | 87.48% |
|  |  | *HDR* | Pp3c23_440V3.1 | 5175 | 16189 | 68.03% |

**Table S3.** The Codon Adaption Index of MEP pathway genes.

| **Species** | **Life lineage** | ***DXS*** | ***DXR*** | ***CMS*** | ***CMK*** | ***MDS*** | ***HDS*** | ***HDR*** |
| --- | --- | --- | --- | --- | --- | --- | --- | --- |
| *A. thaliana* | Eudicots | 0.8 | 0.8 | 0.81 | 0.79 | 0.76 | 0.76 | 0.78 |
| *O. sativa* | Monocots | 0.91 | 0.74 | 0.74 | 0.76 | 0.84 | 0.75 | 0.82 |
| *A. trichopoda* | Flowering plant | 0.74 | 0.78 | 0.76 | 0.81 | 0.76 | 0.77 | 0.8 |
| *P. abies* | Gymnosperm | 0.60 | 0.60 | 0.60 | 0.77 | 0.60 | 0.80 | 0.78 |
| *S. moellendorffii* | Lycophyte | 0.76 | 0.76 | 0.75 | 0.78 | 0.78 | 0.77 | 0.80 |
| *Marchantia polymorpha* | Liverwort | 0.81 | 0.79 | 0.76 | 0.77 | 0.78 | 0.79 | 0.79 |
| *V. carteri* | Green Algae | 0.75 | 0.77 | 0.63 | 0.70 | 0.71 | 0.78 | 0.72 |
| *C. merolora* | Red Algae | 0.84 | 0.84 | 0.83 | 0.84 | 0.84 | 0.85 | 0.85 |
| *Prochlorococcus marinus* | Cyanobacteria | 0.82 | 0.84 | 0.82 | 0.83 | 0.83 | 0.82 | 0.84 |
| *E. coli* | Non-cyanobacteria | 0.77 | 0.72 | 0.69 | 0.65 | 0.71 | 0.76 | 0.79 |
| **Median Value** |  | 0.79 | 0.78 | 0.76 | 0.78 | 0.77 | 0.78 | 0.80 |
